# Two-way messaging therapy for depression and anxiety: longitudinal response trajectories

**DOI:** 10.1186/s12888-020-02721-x

**Published:** 2020-06-12

**Authors:** Thomas D. Hull, Matteo Malgaroli, Philippa S. Connolly, Seth Feuerstein, Naomi M. Simon

**Affiliations:** 1 Teachers College, Columbia University, Talkspace, 33 West 60th Street, 8th Floor, New York, NY 10023 USA; 2 New York University Grossman School of Medicine, New York, USA; 3 Mount Sinai Beth Israel Medical Center, Teachers College, Columbia University, New York, NY USA; 4 Yale University Center for Biomedical and Interventional Technology, New Haven, USA

**Keywords:** Telemedicine, Depression, Anxiety, Longitudinal design, Digital health, Messaging therapy, Text therapy

## Abstract

**Background:**

Telemedicine is a strategy for overcoming barriers to access evidence-based psychotherapy. Digital modalities that operate outside session-based treatment formats, such as ongoing two-way messaging, may further address these challenges. However, no study to date has established suitability criteria for this medium.

**Methods:**

A large outpatient sample (*n* = 10,718) engaged in daily messaging with licensed clinicians from a telemedicine provider. Patients consisted of individuals from urban and rural settings in all 50 states of the US, who signed up to the telemedicine provider. Using a longitudinal design, symptoms changes were observed during a 12 week treatment course. Symptoms were assessed from baseline every three weeks using the Patient Health Questionnaire (PHQ-9) for depression, and the Generalized Anxiety Disorder (GAD-7) for anxiety. Demographics and engagement metrics, such as word count for both patients and therapists, were also assessed. Growth mixture modeling was used to tease apart symptoms trajectories, and identify predictors of treatment response.

**Results:**

Two subpopulations had GAD-7 and PHQ-9 remission outcomes (*Recovery* and *Acute Recovery*, 30.7% of patients), while two others showed amelioration of symptoms (*Depression* and *Anxiety Improvement*, 36.9% of patients). Two subpopulations experienced no changes in symptoms (*Chronic and Elevated Chronic*, 32.4% of patients). Higher use of written communication, patient characteristics, and engagement metrics reliably distinguished patients with the greatest level of remission (*Recovery* and *Acute Recovery* groups).

**Conclusions:**

Remission of depression and anxiety symptoms was observed during delivery of psychotherapy through messaging. Improvement rates were consistent with face-to-face therapy, suggesting the suitability of two-way messaging psychotherapy delivery. Characteristics of improving patients were identified and could be used for treatment recommendation. These findings suggest the opportunity for further research, to directly compare messaging delivery with a control group of treatment as usual.

**Trial registration:**

Clinicaltrials.gov Identifier: NCT03699488, Retrospectively Registered October 8, 2018.

## Background

Anxiety and depression are the leading cause of disability in middle- and high-income nations [1]. Non-pharmacological treatments for these disorders include a variety of evidence-based psychotherapies, which have consistently been found to be effective [2–4]. Nevertheless, access to mental health care is low, leaving many affected individuals untreated [3, 5, 6]. Barriers to care can occur at several levels [7] and include issues such as geographic remoteness, economic or insurance constraints, work or childcare related conflicts, shortage of practitioners, stigmatization, and physical impairment [8–12]. The need to mitigate inequalities of access to care highlights the opportunity for innovative approaches to enhance treatment delivery [13, 14].

Telemedicine interventions offer a solution to increase accessibility, capable of overcoming both geographic and mobility barriers, as well as reducing wait times. A large number of studies have shown that therapy delivered via technology platforms can be effective in symptom reduction, across a range of psychiatric diagnoses [15–20]. Research on technology-mediated treatment has largely been conducted on its most common medium, live video. Newer forms of synchronous and asynchronous delivery have so far received much less attention as a form for delivering direct clinical care [21]. One promising example is two-way multimedia messaging (MMS, or “texting”), given the wide availability of platforms and familiarity with texting as a form of communication. MMS has been effectively used in the past as an adjunct to clinical care, occupying the role of a reminder system or symptom tracker focusing on promoting healthy lifestyle behavior and medication adherence [9, 22, 23]. Synchronous MMS, or “live chat,” has also been used to deliver psychotherapy, and was shown to be effective in combination with a primary care provider [18]. The next generation of MMS treatment has piloted asynchronous modes of delivery, in an attempt to reduce scheduling barriers, expedite treatment initiation, and increase access [21]. In this approach, patients are free to message their provider an unlimited amount 24/7, and clinicians respond during pre-identified times each day for at least 5 days a week. These interactions involve much more text than is typical for a conversation (e.g. between friends or family members), in order to convey the necessary clinical material. Preliminary evidence suggested that this approach may be an acceptable and potentially effective medium for conducting therapy [17, 24]. However, these findings were limited by relatively small sample sizes and retrospective reporting. Further research with larger samples is needed to provide evidence base for asynchronous MMS. If effective, this modality would have the potential to enable more equitable and accessible care by substantially increasing the scale of telehealth.

The current study extends prior findings on asynchronous messaging interventions in two important ways. First, we evaluate the feasibility of wide-scale implementation of asynchronous messaging for delivering therapy within a large provider network, using a longitudinal naturalistic design reflecting the use of this medium in practice [25–27]. Second, the availability of a very large sample enabled the identification of subpopulations within the wider group in order to investigate the heterogeneity of response to the medium in terms of clinical outcome and patient characteristics. This study was designed with a focus on external validity and relevance to clinical practice in the community.

We investigated patterns of response to this novel treatment delivery, and whether large numbers of patients and therapists would find the communication modality feasible. As a result, we did not examine the effectiveness or efficacy of specific types of psychotherapy. Instead, we measured patterns of utilization and dropout, while establishing predictors of response and suitability for the modality. Of particular interest was the number of words and messages sent by patients and by therapists to gauge treatment engagement and dosage, as well as patient characteristics and baseline severity. We report the outcome trajectories that emerged as important exploratory findings for establishing different rates of change and characteristics for this medium [25, 28].

## Methods

### Setting

The study was conducted with a telemedicine platform (Talkspace) used by independently practicing, licensed therapists in the United States. The platform is accessible through internet search, through Employee Assistance Programs, and as a behavioral health benefit through some individual insurances. Patients first meet with an intake clinician through a live messaging system to conduct a brief, standardized intake to identify the presenting complaint, patient treatment history, and the patient’s provider preferences. This information informs a matching algorithm that prioritizes and presents three providers with the desired characteristics for the patient to choose among. Once a clinician is chosen, the provider is alerted, and the patient is immediately introduced to the messaging “room” where treatment takes place. Patients complete a self-report baseline assessment and the provider walks them through the informed consent and emergency contact process after which treatment can begin. Observations in this study include data collected as part of organizational quality assurance and program management processes between January 1, 2016 and February 1, 2018. All patients and clinicians give written consent to the use of their data in a de-identified, aggregate format as part of the user agreement before they begin using the platform. Study procedures were approved as exempt by the institutional review board at Teachers College, Columbia University (15–426).

### Participants

### Patients

Participants were individuals who presented with a chief complaint of anxiety or depression, were seeking treatment through the service, and who completed at least one PHQ-9 and/or GAD-7. Inclusion criteria consisted of: [1] being English speakers in the United States, [2] between the ages of 18 and 65, [3] having regular internet or cellphone access, [4] receiving a depression or anxiety diagnosis from their assigned licensed mental health provider based on a clinical intake and live messaging or video-based interview, as recorded in the electronic medical record with ICD-10 codes, [5] scoring 10 or higher on the PHQ-9 and/or GAD-7. Exclusion criteria consisted of current or past diagnoses of: [1] bipolar disorder, [2] any schizophrenia spectrum and psychotic disorder, or psychotic features, [3] any medical or neurological condition that would better account for the symptoms, [4] substance or alcohol use disorder [5] any condition requiring hospitalization; or [6] suicidal thoughts and/or behavior sufficient to be marked a “Yes” on any of questions three through six (at least thoughts about a potential suicide method), on the Columbia Suicide Severity Rating Scale Lifetime-Recent Screen [29], requiring a more intensive level of care that interrupted treatment on the platform. Twenty three thousand nine hundred one patient records were reviewed with these criteria; the final sample consisted of 10,718 patients.

### Clinicians

Clinicians in the provider network were currently licensed in at least one state, were required to have a Masters degree or above, and had at least 3 years of post-licensure experience delivering mental health care. Clinicians were matched only to patients where licensure included the patient’s residence. There were a total of 1599 clinicians – 43.7% of whom reported five to 9 years of post-licensure experience, and 36.5% reporting ten or more years of experience. Eighty-eight percent (88.0%) were female. Providers had a mean age of 40 (SD = 10.04) years, and as part of their provider profile they reported offering treatment based on multiple orientations: 61.0% cognitive-behavioral treatment, 40.3% third-wave cognitive behavioral interventions (e.g., mindfulness-based), and 25.5% psychodynamic or relational.

### Methods and procedures

#### Intervention

Clinicians and patients asynchronously exchanged text-, audio-, and video-based messages using a secure, HIPAA-compliant platform accessible on mobile devices and on desktop computers. Patients could freely send messages at any time without limit, and all messages were stored for the clinician when they returned to review the message history. Therapists responded to messages from their patients at least once a day, 5 days a week. Clinicians were expected to adhere to all reporting, professional, and ethical standards for their respective fields, and appropriate referrals were provided for patients judged to need a higher level of care.

The number of words exchanged between therapists and patients is automatically counted as meta-data by the platform regardless of the medium, and these counts were used as a proxy to quantify the extent of therapeutic interaction through the asynchronous messaging medium. Words contained within audio and video messages were converted to text to enable word counting using secure and proprietary voice-to-text algorithms. Raw counts of words sent by clinicians and patients were used in supplementary analyses. Raw counts of the number of audio and video messages sent by each party were also analyzed.

#### Assessments

Patients were assessed for depression and anxiety symptoms at baseline and then every 3 weeks for the duration of treatment, or until the patient opted to stop receiving assessments. Assessments are introduced to patients as an important aspect of their care that facilitates goal setting and to track progress. In this study, five assessments from baseline to week 12 were analyzed, including: Baseline, Week 3, Week 6, Week 9, and Week 12.

The 9-item Patient Health Questionnaire [30] was used to identify the clinical severity of depression. Responses on all items were given on a 4-point Likert scale (0 = *Not at all* to 3 = *Nearly every day*) with a total maximum score of 24. Scores greater or equal than 10 have been shown to have high sensitivity and specificity as a threshold for clinical depression, or at least moderate depression [31, 32].

Anxiety symptoms were assessed with the 7-item Generalized Anxiety Disorder questionnaire [33]. Responses on all items were given on a 4-point Likert scale (0 = *Not at all* to 3 = *Nearly every day*) with a total maximum score of 21. Scores of 10 or above have been shown having high sensitivity and specificity as a clinically significant threshold for at least moderate anxiety [34].

Patients opting to leave the platform were asked to indicate the reason for leaving. Reasons included feeling better or meeting their goals, having money concerns, not liking the therapy medium, having frustrating technical issues, not liking their therapist, deciding to continue treatment face-to-face, or no longer having the time necessary to engage in treatment.

### Data analytic strategy

Outcome trajectories of anxiety and depression symptoms over the 12 weeks of treatment were analyzed using Latent Growth Modeling (LGM) in Mplus 8 [35]. LGM is an unsupervised machine learning method to identify groups with heterogeneous outcomes (i.e., such as responders and non-responders) and examine their differences. Compared to traditional average-effects approaches, LGM analyzes patterns of change in the data over time, to determine whether there are subpopulations within the overall group of patients. For example, patients with severe symptoms at baseline who end with low symptoms versus patients that begin and end treatment with a milder symptom presentation. In the current study, LGM also teased patients with changes in both anxiety and depression symptoms, versus those improving in only one of the two conditions. Another advantage to LGM is that once patients have been grouped into different trajectories (or classes), characteristics that are common to each class can be identified (i.e., covariates). For example, patients who share a remission trajectory may be far more likely to be female or engage with treatment more consistently than those in another class. As such, LGM provides much more information in understanding how large groups of people respond to a specific treatment delivery than simply looking at pre- and post-assessment scores for the entire sample. Covariates of interest in this study included age, education, gender, weeks in treatment, words per week for the therapist and words per week for patients. A more technical description of each step of the statistical procedure is provided in the next section.

### Technical specifications of the LGM

Prior to the analyses, missing values for variables with ~ 40% or less missingness [36] were iteratively imputed by random forests (500 trees, 10 iterations), using the R package missForest [37]. Examined predictors were imputed while masking clinical and outcome variables, to prevent information leakage. All LGM models were estimated under missing at random assumptions using maximum likelihood estimation. Sensitivity analysis to assess the relation between missing data in symptoms measures and therapists’ characteristics are reported in the supplementary materials.

To concurrently capture changes in both anxiety and depression outcomes, the LGM modeled concurrent changes of PHQ-9 and GAD-7 scores as parallel processing [35]. Specifically, two sets of distinct intercept, slope, and quadratic growth parameters were assigned to each symptoms measure, estimating separate trajectories of anxiety and depression over five assessments (weeks: 0, 3, 6, 9, and 12). The patients’ classes were then determined based on joint patterns of PHQ-9 and GAD-7 scores growth. The optimal number of classes was determined comparing nested unconditional LGM with increasing numbers of classes. Variance of the growth parameters was fixed to zero, to increase delineation of classes. Examined model fit indices included Bayesian Information Criterion (BIC), sample-size adjusted Bayesian Information Criterion (SSBIC), Akaike Information Criterion (AIC), relative Entropy, Lo–Mendell–Rubin–adjusted likelihood ratio test (L-M-R LRT), and bootstrapped likelihood ratio test (BLRT). The best fitting solution was estimated based on model fit indices, as well as explanatory properties of the solution [38, 39].

After determining the solution with the best relative fit, demographic variables, weeks before treatment dropout (or completion), and therapists’ characteristics were nested as covariates in a conditional LGM, to analyze class membership predictions. Categorical data was subsequently converted into binary variables from modal values. Auxiliary 3-step method multinomial logistic analyses for latent class predictors [40] were then performed on the conditional model. This approach to latent class logistic regression analyses takes into account measurement error in the most likely class attributions, to estimate the predictive role of quantitative treatment delivery characteristics (i.e., the average number of words per week used by therapists and clients over the course of treatment) in determining group membership. Word counts were log-transformed to improve odds ratio dose-response interpretability.

## Results

### Sample characteristics

Patients were between the ages of 18 and 65, with the majority (55.0%) falling between 26 and 35 years of age. Women were 78.9% of the patient sample, and 74.9% of patients had Bachelor’s degrees or higher education level. Table 1 provides the full distribution of demographic and clinical characteristics. Treatment duration was on average 9.75 weeks (SD = 3.16), with 56.24% of the sample completing a 12-week treatment course. Of the 4690 patients discontinuing treatment before 12 weeks, reason for termination was reported by a subset (*N* = 1471, 34% of drop-out): Better/goal met (53.3%), money concerns (22.2%), did not like the treatment medium (10.1%), went to face-to-face treatment (6.9%), technical issues (3.0%), did not like their therapist (2.7%), and no longer had time (2.0%).

**Table 1:** Demographic and Clinical Characteristics for Full Sample (*N* = 10,718).

Variable			# Missing	M (SD) or %
*Age:*			573	
18–25				24%
26–35				55%
36–49				17.9%
50+				3.1%
*Education:*			1549	
Bachelor Degree or Higher				74.9%
High School Diploma				25.1%
*Gender:*			147	
Female				78.9%
Male				21.1%
*Patient’s State:*			1333	
California				15.3%
New York				13.2%
Texas				7.7%
Florida				5.4%
[other U.S. State]				58.4%
*Primary Condition:*			–	
Anx.	Generalized Anxiety Disorder			21.1%
	Anxiety Disorder, Unspecified			16.3%
	Panic Disorder / Agoraphobia			1.5%
Dep.	Major Depressive Disorder, Recurrent Ep.			14.6%
	Major Depressive Disorder, Single Ep.			11.2%
	Mood Disorder, Unspecified			5.0%
	Persistent Depressive Disorder			1.8%
Adj.	Adjustment Disorder with Mixed Anxiety and Depressed Mood			15.3%
	Adjustment Disorder with Anxiety			6.7%
	Adjustment Disorder with Depressed Mood			6.5%
*Reason for Dropout:*			2859	
Feeling Better / Goal Met				53.3%
Money Concerns				22.2%
Didn’t Like Treatment Medium				10.1%
Started face-to-face Treatment				6.9%
Technical Issues				3.0%
Didn’t Like Therapist				2.7%
No Time				2.0%
Treatment Duration (weeks)			–	9.75 (3.16)
*Treatment Engagement:*			2507	
Patient (# words/week)				788.21 (4790.63)
Therapist (# words/week)				626.93 (3556.61)
Working Alliance Inventory			5630	44.84 (10.72)
			**PHQ-9**	**GAD-7**
Observation	# Available	# Dropout	M (SD)	M (SD)
Baseline	10,718	–	13.36 (4.96)	13.35 (4.10)
Week 3	6326	141	9.93 (5.48)	9.76 (4.74)
Week 6	3910	2201	9.04 (5.61)	8.74 (4.79)
Week 9	2264	1684	8.66 (5.66)	8.39 (4.95)
Week 12	1393	484	8.65 (5.96)	8.27 (5.14)

**Fig. 1:**
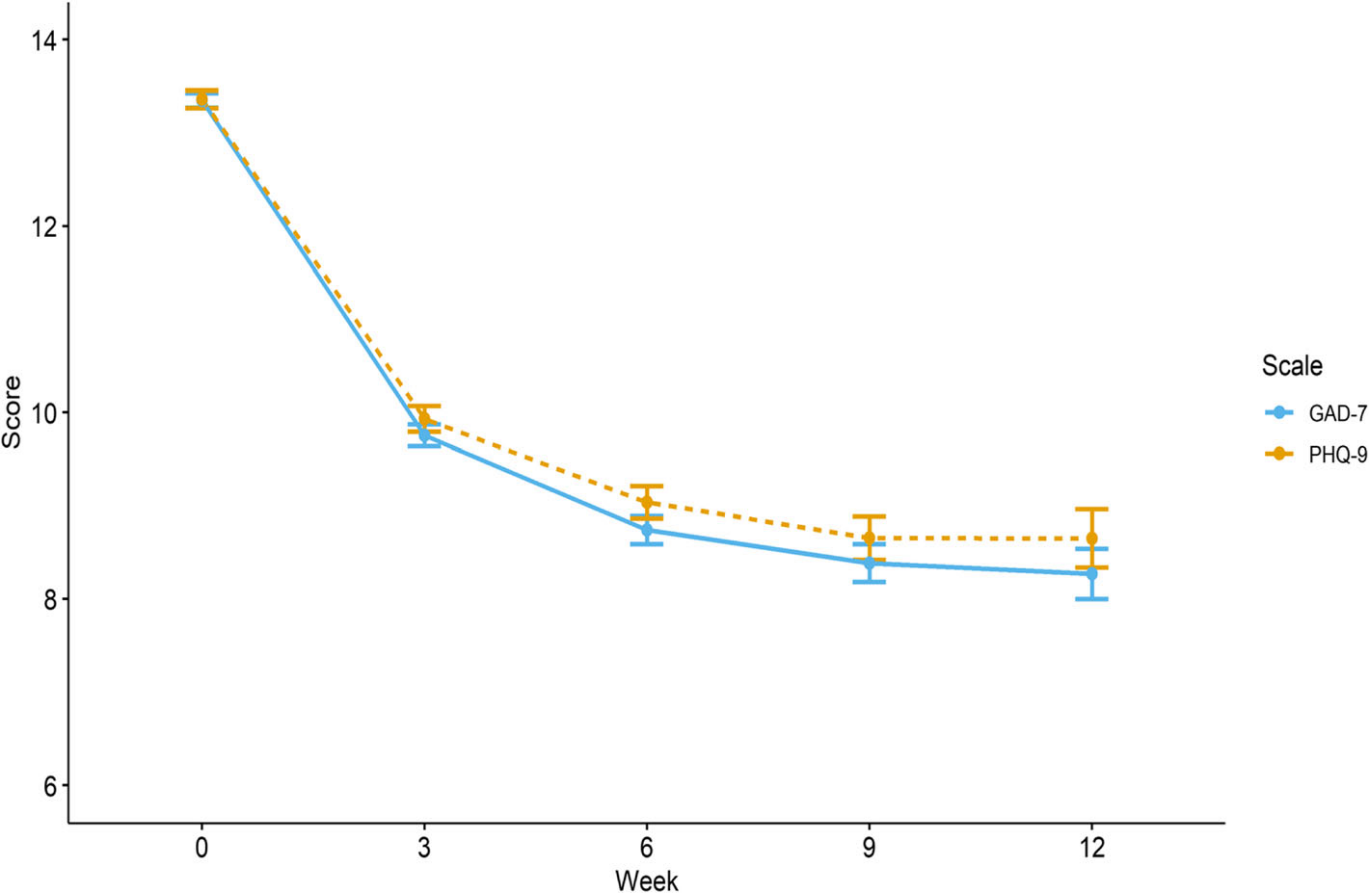
Observed cross-sectional mean PHQ-9 and GAD-7 scores over 12 weeks of two-way messaging treatment (*N* = 10′718). Error bars represent 95% CI. (Dotted line is PHQ-9)

Figure 1 reports overall symptom scores for anxiety and depression at each observation. On average there were 2.30 (SD = 1.32) symptom assessments available per patient. Viewed through the framework of reliable and clinically significant change [41], 53.03% of the sample reported PHQ-9 score reductions of 5 or more points and fell below the established threshold for probable depression, and 47.78% of the sample reported GAD-7 score decreases of 5 or more points and fell below the established threshold for probable anxiety by their last observation. Treatment engagement, as measured by word count, was an average of 788.21 words (SD = 4790.63) per week of treatment generated by patients and 626.93 (SD = 3556.61) by therapists.

### Outcome trajectories of anxiety and depression

Table 2 shows the relative fit indices for progressive model solutions ranging from one to seven classes for parallel latent growth models of PHQ-9 and GAD-7 trajectories. Each of these classes represent a possible subpopulation of patients that is present within the full sample. Model fit indices indicated improved model fit for an increasing number of classes, but the L-M-R LRT did not approach significance for the 7-class solution. The 6-class model was the best fitting and was also judged to have the highest level of interpretability and theoretical utility. Therefore, the 6-class model was chosen as the optimal solution.

**Table 2:** Model Fit Indices for 1 to 7 Classes of Parallel Latent Growth Model of PHQ-9 and GAD-7 Trajectories

	1-class	2-classes	3-classes	4-classes	5-classes	6-classes	7-classes
Akaike Information Criteria	346,037.34	330,711.31	327,235.57	325,672.71	324,545.30	**323,369.51**	322,410.85
Bayesian Information Criteria	346,153.81	330,936.98	327,512.20	326,000.30	324,923.84	**323,799.02**	322,891.31
Sample-Size Adjusted BIC	346,102.97	330,838.46	327,391.44	325,857.30	324,758.59	**323,611.52**	322,681.57
Entropy	–	.79	.72	.71	0.67	**.71**	.71
Lo-Mendell-Rubin Adjusted LRT	–	15,030.40	3436.82	1552.94	1124.11	**1171.74**	957.91
*P*-value	–	*<.001*	*<.001*	*.018*	*<.001*	***<.001***	*.264*
Bootsrapped LRT P-value	–	*<.001*	*<.001*	*<.001*	*<.001*	***<.001***	*<.001*

**Fig. 2:**
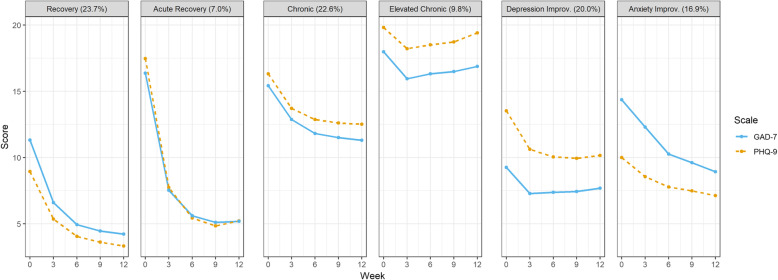
Parallel Growth Trajectories of PHQ-9 and GAD-7 estimated scores. Each Class is teased based on their longitudinal course of both depression and anxiety symptoms over 12 weeks of treatment (*N* = 10,718). (Dotted line is PHQ-9)

The best fitting parallel Latent Growth Model is displayed in Fig. 2. The model identified six subpopulations that were differed in their change of depression and anxiety symptoms over 12 weeks of treatment. Probability of distinct class membership for each individual participant was high, with values ranging from .75 to .86. The most common class showed improving GAD-7 and PHQ-9 symptoms scores (*Recovery*, 23.7%). This group was characterized by moderate levels of anxiety and mild depression symptoms at baseline, which steadily lowered over treatment and remained at subclinical levels. A more rapid recovery pattern was displayed by another class (*Acute Recovery,* 7.0%). This class displayed initial high symptomatology for both anxiety and depression, which sharply decreased below clinical thresholds over the course of treatment. Two additional classes presented with high scores for both GAD-7 and PHQ-9; symptoms in these groups only marginally improved (*Chronic*, 22.6%) or remained elevated (*Elevated Chronic*, 9.8%) through therapy. As such, these two subpopulations did not respond to treatment. Two other remaining classes (*Depression Improvement*, 20.0%; *Anxiety Improvement*, 16.9%) presented with moderate symptoms of depression and anxiety, which improved into milder severity over the course of treatment.

### Predictors of symptoms remission

Conditional LGMs analyzed the role of patients’ covariates in predicting remission outcomes (*Recovery* and *Acute Recovery* group). Gender, age, education, treatment length, and therapist characteristics (years of experience and expertise) were nested as covariates in the model, while the number of words used by therapist-patient dyads every week were included as predictors using 3-step auxiliary analysis. There were no substantial changes in the shape and proportions of the trajectories from the unconditional solutions. Symptoms trajectories adjusted for the covariates and their individual patients scattering are reported in the supplementary materials.

Of note, 3-step latent class logistic regression analyses indicated significant differences in the use of the messaging service between trajectory classes. Patients assigned to both Recovery groups were more likely to engage in weekly written communication with their therapist compared to all other classes, while controlling for age, education, gender, and treatment duration. Moreover, the therapists of individuals in non-Recovery groups were more likely to try to engage their patients using written communication, resulting in higher average therapist words generated per week. The *Acute Recovery* class was differentiated from the other *Recovery* group by higher likelihood of treatment adherence and lower education; nevertheless, the two Recovery classes did not significantly differ between them by amount of words used with their therapist or received by their therapists.

Results from the multinomial logistic regression analyses indicated that, when compared to the recovery groups, all other patients had lower treatment durations and thus were less likely to complete treatment. When specifically compared to the largest *Recovery* group, all other classes had less likelihood of having a college degree or higher – with the exception of the *Anxiety Improvement* group. This latter group was also more likely to have higher education levels than the patients in the *Acute Recovery* group which in turn had more likelihood of having a therapist who self-identified with a CBT approach. *Acute Recovery* patients were also more likely to have a self-identified CBT therapist and to have at least a bachelor degree when compared to the *Elevated Chronic* group. Table 3 reports the full estimates and their confidence intervals.

**Table 3:** Multinomial Logistic Regression for predictors of PHQ-9 and GAD-7 trajectories class memberships (N = 10,718)

	*Ref: Recovery*																*Ref: Acute Recovery*										
	Acute Recovery			Chronic			Elevated Chronic			Depression Improv.			Anxiety Improv.			Chronic			Elevated Chronic			Depression Improv.			Anxiety Improv.		
Variable	OR	95% CI	*P*	OR	95% CI	*P*	OR	95% CI	*P*	OR	95% CI	*P*	OR	95% CI	*P*	OR	95% CI	*P*	OR	95% CI	*P*	OR	95% CI	*P*	OR	95% CI	*P*
Age (≥36 Years)	1.11	0.83–1.49	.436	0.93	0.71–1.2	.377	1.07	0.81–1.41	.578	1.01	0.78–1.29	.955	0.82	0.64–1.06	.182	0.84	0.64–1.09	.186	0.96	0.73–1.28	.80	0.91	0.7–1.18	.47	0.74	0.54–1.01	.06
Gender (Female)	1.14	0.87–1.51	.324	**1.25**	**0.96–1.63**	**.014**	0.89	0.68–1.18	.29	0.93	0.72–1.21	.462	0.99	0.75–1.29	.892	1.09	0.83–1.44	.531	0.78	0.59–1.04	.091	0.82	0.62–1.07	.139	0.86	0.65–1.15	.311
Education (Bachelor+)	**0.55**	**0.42–0.72**	**<.001**	**0.59**	**0.46–0.75**	**<.001**	**0.37**	**0.28–0.47**	**<.001**	**0.58**	**0.45–0.74**	**<.001**	0.88	0.69–1.13	.354	1.07	0.82–1.39	.627	**0.67**	**0.51–0.86**	**.002**	1.05	0.81–1.35	.72	**1.61**	**1.2–2.15**	**.001**
Weeks in Treatment	**1.09**	**1.05–1.14**	**<.001**	**0.95**	**0.91–0.99**	**.001**	**0.92**	**0.88–0.96**	**<.001**	**0.93**	**0.9–0.97**	**<.001**	**0.93**	**0.89–0.97**	**<.001**	**0.87**	**0.83–0.91**	**<.001**	**0.84**	**0.8–0.88**	**<.001**	**0.85**	**0.82–0.89**	**<.001**	**0.85**	**0.81–0.89**	**<.001**
Words per Week: Patient	1.08	0.89–1.30	.451	**0.70**	**0.61–0.81**	**<.001**	**0.66**	**0.55–0.78**	**<.001**	**0.67**	**0.57–0.79**	**<.001**	**0.73**	**0.61–0.86**	**<.001**	**0.65**	**0.54–0.80**	**<.001**	**0.61**	**0.49–0.75**	**<.001**	**0.62**	**0.51–0.76**	**<.001**	**0.68**	**0.55–0.84**	**<.001**
*Therapist:*																											
Years of Experience	1.03	0.77–1.37	.743	1.10	0.85–1.42	.066	1.06	0.8–1.39	.36	1.06	0.82–1.36	.332	1.02	0.79–1.32	.745	1.07	0.91–1.25	.405	1.03	0.88–1.21	.717	1.03	0.88–1.2	0.713	1.00	0.85–1.17	.947
Orientation: CBT	1.19	0.9–1.57	.166	1.10	0.84–1.43	.251	0.84	0.64–1.11	.067	0.93	0.72–1.21	.434	0.91	0.69–1.18	.309	0.92	0.71–1.2	.549	**0.70**	**0.54–0.92**	**.009**	0.78	0.61–1.01	0.061	**0.76**	**0.58–0.99**	**.044**
Orientation: Third Wave	0.90	0.69–1.19	.404	1.00	0.78–1.27	.96	0.97	0.76–1.25	.785	1.06	0.83–1.36	.497	0.95	0.74–1.22	.611	1.10	0.86–1.41	.443	1.08	0.83–1.39	.583	1.17	0.92–1.49	0.196	1.05	0.82–1.36	.698
Orientation: Dynamic	1.16	1.11–1.21	.391	1.00	0.96–1.04	.99	0.96	0.92–1.01	.799	0.94	0.9–0.98	.631	1.19	1.14–1.25	.189	0.86	0.61–1.23	.418	0.83	0.58–1.2	.326	0.81	0.57–1.15	0.236	1.03	0.73–1.46	.86
Words per Week: Therapist	1.15	0.90–1.48	.252	**1.69**	**1.40–2.03**	**<.001**	**2.21**	**1.76–2.79**	**<.001**	**1.77**	**1.44–2.19**	**<.001**	**1.83**	**1.47–2.27**	**<.001**	**1.46**	**1.12–1.90**	**.005**	**1.92**	**1.44–2.55**	**<.001**	**1.54**	**1.18–2.00**	**.005**	**1.58**	**1.21–2.07**	**.001**

Note. *Ref* Reference Class; *OR* Odds Ratio; 95% *CI* 95% Confidence Interval

## Discussion

This study examined outcome trajectories following 12 weeks of psychotherapy delivered through asynchronous two-way messaging. The study involved a very large sample of treatment seeking individuals with clinician-reported diagnoses of depression or anxiety, endorsing symptoms in the moderate to severe range. Results showed that depression and anxiety symptoms decreased in the majority of the identified subpopulations (67.6% of the sample), with nearly a third reporting very few symptoms indicating a good outcome relative to the established thresholds of the measures (*Recovery* and *Acute Recovery*, 30.7% below the mild threshold). Clinically significant symptoms improvements were observed in 47.78% of the sample for GAD-7 and in 53.03% for PHQ-9. Nevertheless, the remaining Chronic groups (32.4% of the total sample) endorsed elevated symptoms throughout treatment. The six identified patient outcome trajectories were distinguished by baseline severity, rates of improvement, education level, treatment adherence, and by number of words generated by therapist and patient. In particular, both Recovery groups were associated with higher written engagement during treatment compared to the other groups. These differences could reflect patient characteristics (e.g., greater motivation and treatment readiness), therapist characteristics (e.g., greater interpersonal skills, warmth, and experience with evidence supported interventions for the presenting complaint) or a combination of the two. Patients assigned to the Chronic and Improvement groups also tended to receive more messages than both *Recovery* and *Acute Recovery* groups, suggesting that therapists may have had to work harder to keep them engaged in treatment or to work through complex challenges; for patients in the *Chronic* and *Elevated Chronic* groups, psychotherapy could have served to maintain their condition. Importantly, the contrast in the number of words exchanged among groups with different outcomes is potentially instructive about the mechanisms of change in this medium; however, further research is needed to investigate the content and frequency of the messaging based interchanges.

In terms of treatment length, the majority of the sample (59.1%) adhered to treatment for the entirety of the 12 weeks of messaging therapy. In particular, patients in the *Acute Recovery* groups had the highest treatment completion likelihood. Available data on reasons for dropout highlighted that termination in these groups was also due to treatment goal completion (53.3%), an important consideration when gauging the acceptability of any form of treatment [25]. Overall, messaging treatment adherence compared favorably to face-to-face interventions, where the modal number of sessions attended in traditional settings is one, with a median of five sessions [42]. The notable increase in treatment length is likely to be accounted for by the accessibility afforded by two-way messaging, which allows patients to asynchronously communicate with their therapist whenever is most convenient and from any location. It is also interesting to note that patients who had an *Acute Recovery* of symptoms from an elevated baseline were significantly more likely to have a therapist who self-reported a CBT orientation than patients whose symptoms remained *Elevated Chronic* throughout the study. A higher likelihood of CBT was also observed when compared to patients who only had moderate improvement in anxiety.

It is possible that these outcome differences reflect the adaptability and non-inferiority of delivering CBT through multiple digital formats [16]. A less informative possibility is that this finding reflects an artifact given how many therapists reported a CBT orientation, or may indicate something about therapists who self-identify with a CBT orientation, rather than whether CBT practice was in fact used more by these therapists, a variable that was not measured in this study. However, no other expertise differences or therapist characteristics (beyond texting engagement) emerged as significant when comparing patients to those acutely or moderately improving. Future studies using messaging delivery should quantify interventions for the medium and assess their content beyond self-reported clinical orientation.

An important feature of the patients represented in this study is that large proportions of the sample are well-educated (75% with a Bachelor’s degree or higher) and female (78%). This may be an artifact of using a convenience sample that is driven by advertising practices and the channels used to promote adoption of the service, than a statement of suitability for any particular population. However, the high proportion of female participants is consistent with data for telemedicine in routine care, whereas the education level in this sample is higher than that previously reported [43]. Research that investigates outcomes for more broadly representative samples will help to resolve the interesting issue of whether messaging therapy is acceptable to a wide variety of demographic groups.

The current study reflects messaging telemedicine in practice, observing a very wide range of patients across the United States, from both rural and urban settings, over time. Despite these strengths, the study’s research design also presented limitations. In particular, examining response trajectories without a control group is limited in its ability to determine the relative effectiveness of messaging therapy, or to control for historical factors and spontaneous remission. However, the LGM analyses address criticisms of regression to the mean and capitalizing on mild cases of depression. In the first case, regression to the mean is a statistical effect within homogenous populations, whereas LGM teases apart and identifies each subpopulation and models change over time for that subpopulation [42]. In the second case, the LGM identified that while there was a population presenting with mild depression, there were other populations as well that reported greater severity at baseline. Nevertheless, future studies with a control arm and /or an active standard of care comparator, and randomized patient assignment would be an important complement for fully testing the efficacy of treatment delivery on the messaging medium.

Other limitations of this study include missing post-baseline assessments for some patients, and no content analyses to supplement word count for further clarifying subpopulation differences and reasons for response or non-response. Future studies might also consider more extensive assessments such as structured diagnostic interviews, and quality of life and function measures; however, setting up the study as a clinical efficacy trial, depending on the demands, could diminish generalizability. Lastly, the subpopulations identified may only generalize to telemedicine settings that use two-way asynchronous messaging for treatment delivery.

Notwithstanding the limitations above, these results complement findings from early research on treatment via messaging [17, 23], and extend these findings with a much expanded sample size, the use of longitudinal assessment, and by identifying patient characteristics likely to benefit from therapy in this medium as it is practiced in the field [26, 27]. Further investigation into mechanisms of therapeutic action could greatly enrich our understanding of this medium.

## Conclusions

There is growing interest in and utilization of modern communication media for treatment interventions, with uptake at a rapid pace ahead of formal research. It is thus critical to examine these forms of intervention to determine their effectiveness and understand who may benefit and be appropriate for this type of care. We identified two response groups for patients with moderate to severe depression and/or anxiety that achieved symptom remission, as well as two groups with symptom improvement. Several prognostic factors and patient characteristics were identified that predicted whether a patient is likely to experience remission that are critical for evaluating the impact of novel treatment modalities. Dropout rates also suggested that this medium affords added convenience that enables patients to continue with treatment for a longer duration than is reported in traditional settings [44].

## Supplementary information

**Additional file 1: Supplementary Table 1.** Logistic regression of therapists’ characteristics as predictors of PHQ-9 and GAD-7 missing data. **Supplementary Table 2.** Missing values per most likely categorical LGM class assignment. **Supplementary Fig. 1.**. Covariate adjusted estimated means of PHQ-9 and GAD-7 for recovery and chronic classes, with observed individual trajectories.

## Data Availability

The datasets used and/or analyzed during the current study are available from the corresponding author on reasonable request.

## Abbreviations

MMS: Multimedia message service
PHQ-9: Patient health questionnaire – 9
GAD-7: Generalized anxiety disorder – 7
ICD-10: International classification of diseases − 10
HIPAA: Health insurance portability accountability act
LGM: Latent growth modeling
