# Supplementary material for: Two-way messaging therapy for depression and anxiety: longitudinal response trajectories
Source: BMC Psychiatry. 2020 Jun 12;20:297. doi: 10.1186/s12888-020-02721-x (PMC7291694; doi:10.1186/s12888-020-02721-x)

**Sensitivity Analyses.**

All statistical analyses were performed under Missing at Random (MAR) assumptions. Sensitivity analysis were conducted to assess MAR, and focused on therapists’ characteristics as reported on their provider profile: years of experience, self-reported CBT orientation, self-reported psychodynamic orientation, and self-reported third wave (e.g., DBT) orientation. Firstly, Little’s Missing Completely at Random (MCAR) test was performed to test MCAR assumptions by including all survey measures and therapists experience and expertise. Results from the test (χ^2^(86, 10718) = 110.98; *P*=.036) confirmed assumptions that the data does not fit MCAR. Secondly, a logistic regression analysis was performed to further assess the association between therapist characteristics and missingness. A dichotomic missingness variable indicator was created based on non-response to one or more clinical surveys (i.e. PHQ-9 and GAD-7) over the course treatment. Interaction effects were included in the analysis between self-reported treatment orientation and reported years of experience categorized in three groups: less than five, between 5-9, and ten or more years of experience. Results from the logistic regression analysis suggest that no significant association emerged between missing data, therapists’ variables and their interaction effects (χ^2^ (5, 10718) = 12.36); *P*=337; McFadden Pseudo R^2^= .002). Supplementary Table 1 reports the full estimates.

**Supplementary Table 1.**

Logistic regression of therapists’ characteristics as predictors of PHQ-9 and GAD-7 missing data.

| Variable | | OR | | *P* | | | 95% CI | |
| --- | --- | --- | --- | --- | --- | --- | --- | --- |
| (Intercept) |  | 12.745 |  |  | <.001 |  | | [2.23; 2.86] |
| *Experience: (Ref: Exp. <5yr)* |  |  |  |  |  |  | |  |
| *5yr ≤ Exp. <10* |  | 0.840 |  |  | 0.342 |  | | [-0.53; 0.19] |
| *Exp. ≥ 10* |  | 0.792 |  |  | 0.217 |  | | [-0.60; 0.14] |
| Self-reported Orientation: CBT |  | 0.836 |  |  | 0.347 |  | | [-0.55; 0.19] |
| Self-reported Orientation: Psychodynamic |  | 0.822 |  |  | 0.308 |  | | [-0.57; 0.18] |
| Self-reported Orientation: Third Wave |  | 0.962 |  |  | 0.827 |  | | [-0.39; 0.31] |
| *Interactions:* |  |  |  |  |  |  | |  |
| 5yr ≤ Exp. <10 * CBT |  | 0.969 |  |  | 0.888 |  | | [-0.47; 0.40] |
| 5yr ≤ Exp. <10 * Psychodynamic |  | 1.037 |  |  | 0.872 |  | | [-0.40; 0.48] |
| 5yr ≤ Exp. <10 * Third Wave |  | 1.060 |  |  | 0.781 |  | | [-0.35; 0.47] |
| Exp. ≥ 10 * CBT |  | 1.072 |  |  | 0.755 |  | | [-0.37; 0.51] |
| Exp. ≥ 10 * Psychodynamic |  | 1.192 |  |  | 0.440 |  | | [-0.27; 0.62] |
| Exp. ≥ 10 * Third Wave |  | 1.107 |  |  | 0.636 |  | | [-0.32; 0.52] |
| *Note.* Ref. = Reference group*;* Exp. = Years of Experience*;*  OR = Odds Ratio; 95% CI = 95% Confidence Interval. | | | | | | | | |

Thirdly, examination of missing data patterns per trajectory class membership are reported in Supplementary Table 2. Chi Square analysis showed significant differences between the classes (χ^2^(5, 10718) = 58.6); *P<*.001) survey adherence. Specifically, the *Acute Recovery* and *Recovery* group had the least missing data compared to the others classis. These findings were consistent with overall treatment adherence and dropout examination from the conditional analysis in the main manuscript.

**Supplementary Table 2.**

Missing values per most likely categorical LGM class assignment.

| Class | Missing, N(%) | |
| --- | --- | --- |
|  | None | 1+ |
| Acute Remission | 113 (17%) | 555 (83%) |
| Anxiety Improvement | 159 (9%) | 1582 (91%) |
| Chronic | 228 (9%) | 2300 (91%) |
| Depression Improvement | 182 (8%) | 2044 (92%) |
| Elevated Chronic | 82 (8%) | 925 (92%) |
| Remission | 293 (11%) | 2255 (89%) |

Lastly, the GAD-7 and PHQ-9 parallel growth model was adjusted using a nested conditional approach. Patients’ characteristics (i.e., age, education, gender, weeks in treatment) and therapist characteristics (i.e. years of experience and self-reported CBT, third wave, and psychodynamic orientation) were included in a nested conditional model based on the best fitting unconditional solution. The resulting maximum likelihood estimation of PHQ-9 and GAD-7 growth leveraged information from differences in covariate between classes to adjust the trajectories. Compared to the unconditional model, the adjusted conditional solution presented similar classification quality (Entropy = .71), range of average class probabilities (.75-.86), and similar group membership proportions: *Recovery* (22.8%), *Acute Recovery* (7.2%), *Chronic* (22.9%), *Elevated Chronic* (10.5%), *Depression Improvement* (20.4), and *Anxiety Improvement* (16.3%). Odds ratios for class and conditional model variables are further reported in the main manuscript. Trajectories solutions for remitting and non-remitting groups, along with individual outcomes of group members (i.e., spaghetti plot) are reported in Supplementary Figure 1.

Supplementary Fig. 1. Covariate adjusted estimated means of PHQ-9 and GAD-7 for recovery and chronic classes, with observed individual trajectories.


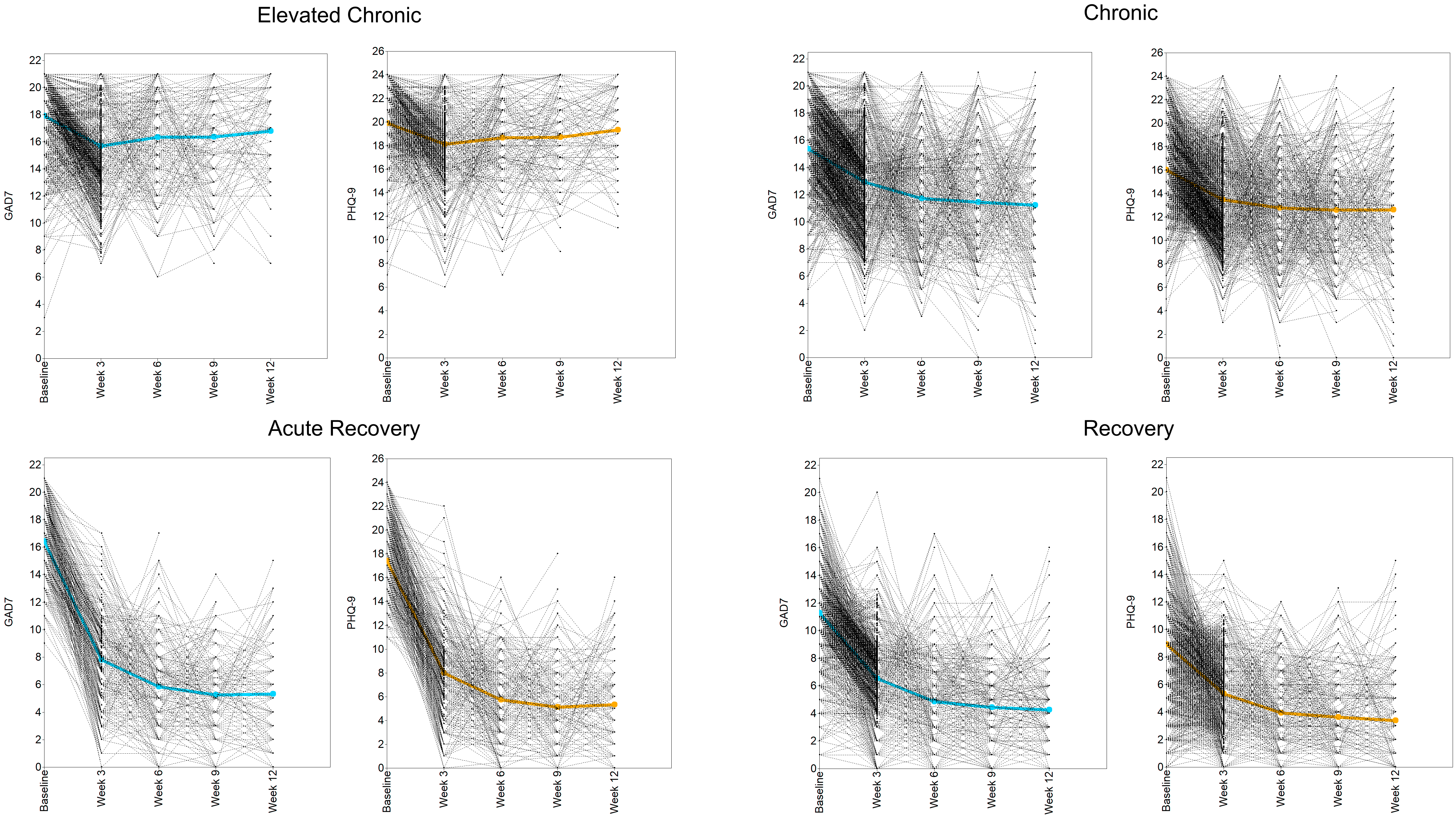

Supplement: Supplementary file 1 — Additional file 1: Supplementary Table 1. Logistic regression of therapists’ characteristics as predictors of PHQ-9 and GAD-7 missing data. Supplementary Table 2. Missing values per most likely categorical LGM class assignment. Supplementary Fig. 1.. Covariate adjusted estimated means of PHQ-9 and GAD-7 for recovery and chronic classes, with observed individual trajectories. [file 12888_2020_2721_MOESM1_ESM.docx]
